# What does not kill you makes you stronger: surviving anti-cancer therapies by cytoskeletal remodeling and Myosin II reactivation

**DOI:** 10.1080/23723556.2020.1735911

**Published:** 2020-03-26

**Authors:** Jose L Orgaz, Victoria Sanz-Moreno

**Affiliations:** a Barts Cancer Institute, Queen Mary University of London, John Vane Science Building, London, UK; b Facultad de Ciencias Experimentales, Universidad Francisco de Vitoria, Madrid, Spain

**Keywords:** Myosin II, cytoskeletal remodeling, melanoma therapy resistance, transcriptional rewiring, immunotherapy

## Abstract

Myosin II and its regulator Rho-associated coiled-coil containing protein kinase (ROCK) are essential for cell invasion and metastatic dissemination. Our recent findings show that this molecular machinery is also involved in drug resistance in melanoma by playing a dual role: protection of tumor cells from reactive oxygen species (ROS) and DNA damage (intrinsic), and co-option of myeloid and lymphoid populations to establish immunosuppression (extrinsic).

Non-muscle Myosin II (Myosin II hereafter) is a holoenzyme with actin cross-linking and contractile properties. Myosin II-driven cell contractility relies on Rho GTPase signaling that through Rho-associated coiled-coil containing protein kinase (ROCK) increases phosphorylation of myosin light chain 2 (p-MLC2) and Myosin II activity.^1^ Myosin II activation generates contractile force essential for several cellular processes, including cytokinesis, force-mediated matrix remodeling and, critically, migration, and metastatic dissemination.^2^ We have recently found that apart from these cell-intrinsic functions, ROCK-Myosin II can shape the microenvironment and polarize macrophages to tumor-promoting phenotypes (CD206+).^3^

Metastasis is responsible for most deaths of cancer patients. While there are some therapies that achieve impressive responses and extend patient survival, these are not long-lasting due to drug resistance, both intrinsic/primary and acquired. This is exemplified in cutaneous melanoma, a highly aggressive and metastatic skin cancer. Most melanoma patients harbor mutations in the mitogen-activated protein kinase (MAPK) pathway (BRAF-MEK-ERK) (*BRAF*^V600E^ being the most common) and therefore, benefit from targeted therapies against MAPK pathway (BRAF and MEK inhibitors). However, resistance inevitably arises in most patients within a year, in most cases due to ERK restoration.^4^

Immunotherapies against immune checkpoints programmed cell death protein 1 (PD-1) and cytotoxic T-lymphocyte-associated protein 4 (CTLA-4), aimed to awake the immune system that will eliminate the tumor, has proven successful in a subset of melanoma patients. However, response rates are low (less than 40%) and a substantial proportion of responders relapse within 2 years.^5^ Therefore, metastasis and drug resistance are major problems that limit achieving long-lasting cures in melanoma patients.

The last decade has seen a myriad of efforts in deciphering mechanisms of resistance to targeted and immunotherapies in melanoma.^4^ Interestingly, cross-resistance to both MAPK inhibitors (MAPKi) and immune checkpoint blockers (ICB) is driven by common alterations at the transcriptional level in regulators of metastatic features (invasion, epithelial–mesenchymal transition (EMT), extracellular matrix (ECM) remodeling).^6,7^ Therefore, in our latest study, we assessed if Myosin II and cytoskeletal remodeling could play a role in therapy-cross-resistance.^8^

Rho GTPase signaling and cytoskeletal remodeling were top enriched processes in the phospho-proteome of melanoma cells after a 24 h treatment of melanoma cells with MAPKi. MAPK inhibition decreased Myosin II activity (p-MLC2 levels), regardless of genetic background (BRAF mutant, BRAF mutant/PTEN-null, NRAS mutant). Surprisingly, 48 h after MAPK inhibition Myosin II levels were fully restored or even increased, while ERK activity was only partially recovered. This uncoupling of Myosin II from MAPK suggested that Myosin II could confer a survival advantage under therapy. In fact, overexpression of constitutively active MLC2 conferred resistance to BRAFi. Therefore, under the selective pressure of treatment, Myosin II uncouples itself from MAPK signaling to provide a survival advantage.

We next wondered if restoration of Myosin II in resistant cells could become a new vulnerability. BRAFi-resistant cells were much more sensitive to Myosin II blockade using ROCK inhibitors (ROCKi) or ROCK1/2 RNAi. This increased sensitivity was observed in 2D and 3D environments in a panel of several models of drug resistance (BRAFi-, BRAFi+MEKi-resistant cell lines, and patient-derived lines). Direct inhibition of Myosin II with a specific inhibitor (blebbistatin) and knockdown of either Myosin II heavy chain (*MYH9*) or light chains (*MYL9/12B*) with RNAi impaired survival of resistant cells. At this point, the prediction was that cross-resistant melanomas, having transcriptionally rewired their cytoskeleton, would be refractory to a second therapy (i.e., immunotherapy) but now much more sensitive to Myosin II blockade. In fact, anti-PD-1-resistant cell lines were more sensitive to Myosin II inhibition with ROCKi, suggesting that this vulnerability is an intrinsic feature of therapy-resistant cells regardless of the therapy. Survival of patient-derived cell lines resistant to sequential MAPKi+ICB was also impaired upon Myosin II inhibition, indicating some level of cross-resistance.

To assess the clinical relevance of these findings, we analyzed published transcriptomes of resistant patients. Half of the patients (on-MAPKi, MAPKi-resistant, and on-anti-PD-1-resistant) upregulated ROCK-Myosin II pathway genes. Melanomas resistant to anti-PD-1 had also higher Myosin II levels before therapy, suggesting its potential as a biomarker of lack of response. In fact, in The Cancer Genome Atlas (TCGA) melanoma database we found that higher expression of ROCK-Myosin II pathway correlates with poorer prognosis, indicating that higher Myosin II levels before therapy could identify more aggressive melanoma cells that would have a survival advantage later on under therapy.

When we analyzed 12 paired samples from patients before and after therapy (MAPKi, ICB, or sequential MAPKi+ICB), using immunohistochemistry (IHC) we measured increased p-MLC2 levels in the resistant samples. This was accompanied by changes in the tumor microenvironment: increased matrix deposition (which could contribute to higher p-MLC2) and higher numbers of immunosuppressive populations, in particular CD206+ macrophages and forkhead box P3 (FOXP3+) regulatory T cells (Tregs). This tumor-supporting microenvironment could explain therapy failure (Figure 1). It will be important to validate these IHC results in a larger cohort of patients.

**Figure 1.**
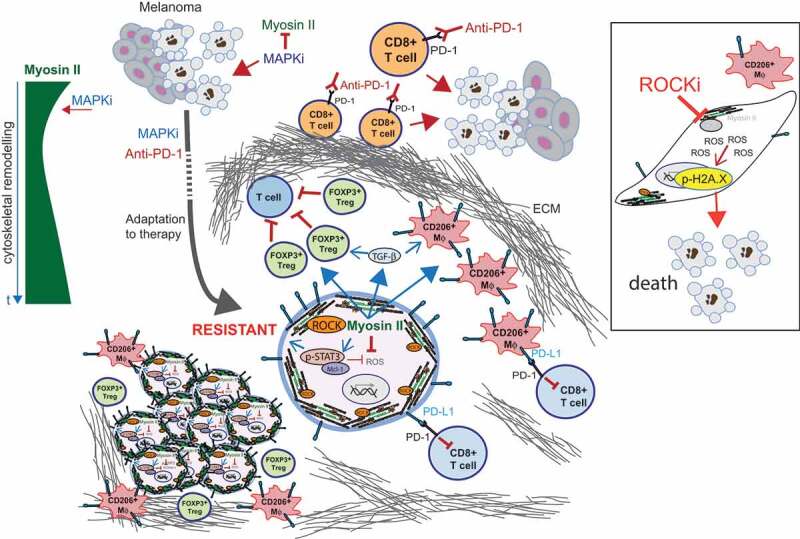
Novel roles of ROCK-Myosin II in therapy resistance. Left, diagram showing Myosin II levels after MAPKi treatment. Myosin II is initially reduced but, over time (t), there is profound cytoskeletal remodeling and Myosin II levels are restored. Middle, therapies (MAPK inhibitors (MAPKi); Anti-programmed cell death protein 1 (PD-1)) initially induce melanoma tumor regression. However, during adaptation to therapy surviving cells remodel their cytoskeleton and reactivate Myosin II. In resistant cells, Rho-associated coiled-coil containing protein kinase (ROCK)-Myosin II enhance survival by increasing pro-survival signaling (phosphorylated signal transducer and activator of transcription 3 (p-STAT3)-Mcl-1), thereby lowering reactive oxygen species (ROS) and DNA damage (intrinsic effects). High p-STAT3 could also contribute to higher levels of PD-1 ligand PD-L1. ROCK-Myosin II also supports an immunosuppressive microenvironment by promoting forkhead box P3 (FOXP3+) regulatory T cells (Tregs), most likely due to increased transforming growth factor beta (TGF-β;) and by polarizing macrophages (Mφ, CD206+, PD-L1+) (extrinsic effects). Resistant tumors present increased extracellular matrix (ECM) deposition, which could also hamper access of therapies and/or T cells. Right, inhibition of ROCK-Myosin II with a ROCK inhibitor (ROCKi) induces lethal ROS levels and increased DNA damage, leading to cell death. In addition, ROCKi reduces FOXP3+ Treg numbers and also PD-L1 expression in CD206+ macrophages, relieving immunosuppression.

Next, we investigated the mechanism underlying the increased dependency of resistant cells on Myosin II. We had previously shown that lowering ROCK-Myosin II increased reactive oxygen species (ROS) in migrating melanoma cells.^9^ We found that MAPKi-resistant cells harbor deregulated ROS metabolism and defective DNA damage repair. We exploited these vulnerabilities and found that ROCKi induced high ROS, DNA damage, and a pronounced cell cycle arrest in BRAFi-resistant cells compared to the sensitive counterpart. ROCKi reduced pro-survival signals mediated by phosphorylated signal transducer and activator of transcription 3 (p-STAT3)-Mcl-1. All these combined effects led to cell death (Figure 1).

We then tested ROCKi in pre-clinical therapy-resistant mouse models. Combination of ROCKi with BRAFi reduced growth of BRAFi-resistant melanoma xenografts in nude mice. This could be due to increased intrinsic cell death induced by ROCKi in resistant cells. Furthermore, ROCKi-treated tumors had reduced p-MLC2 and lower numbers of CD206+ macrophages (total number of F4/80+ macrophages were unaffected), which could also contribute to reduced tumor growth in an extrinsic manner. Importantly, using an experimental metastasis assay, we found that pre-treatment with ROCKi impaired survival of BRAFi-resistant patient-derived melanoma cells in the lung. Altogether, these data suggest that ROCKi could be used to impair both primary tumor growth and metastatic dissemination of resistant melanoma cells.

We next investigated if ROCKi could enhance ICB efficacy. Indeed, combination of ROCKi with anti-PD-1 induced more tumor regressions than single anti-PD-1. ROCKi relieved immunosuppression by decreasing CD206+ macrophages and FOXP3+ Tregs. We have previously described that transforming growth factor beta (TGB-β), a potent immunosuppressor, promotes Myosin II-driven contractility in melanoma.^10^ Interestingly, ROCKi reduced TGB-β levels in immunotherapy-resistant cells *in vitro*, which could lead to dampening of immunosuppression *in vivo*.

Similar to the human setting, we observed variable responses to anti-PD-1 treatment in mice; therefore, we isolated a non-responder that was allografted into new recipient mice. In this model of anti-PD-1 resistance, cancer cells had increased Myosin II and had polarized all macrophages to CD206+ phenotype. Importantly, combination ROCKi+anti-PD-1 induced more tumor regressions and reduced numbers of FOXP3+ Tregs. Furthermore, ROCKi+anti-PD-1 decreased levels of PD-1 ligand (CD274, best known as PD-L1) in both tumor cells and CD206+ macrophages (Figure 1), which could contribute to enhanced anti-PD-1 efficacy. Macrophages are one of the main sources of PD-L1 in the melanoma microenvironment^11^ and, therefore, immunosuppression. In fact, anti-PD-1/PD-L1 therapies have been suggested to function also directly on macrophages.^12^

Our study shows that adaptation to therapy (targeted, ICB) and development of resistance comes with a cost, since it involves profound cytoskeletal remodeling and Myosin II reactivation. Resistant cells, therefore, gain a new vulnerability, which can be exploited by targeting Myosin II with ROCKi (Figure 1). Given the vasodilator effects of ROCKi,^1^ further pre-clinical studies assessing dose-escalation, treatment schedule, and delivery modality (local, antibody–drug conjugate) will be needed in order to optimize ROCKi. Such studies will aim to assess superior responses of ROCKi as single therapy, or increase efficacy when combined with standard of care therapies. Our study raises the possibility that other MAPK-driven tumors and ICB-resistant cancers could be vulnerable to ROCK-Myosin II inhibition, which warrants further investigation.
